# Ten ways to make the most of World Antimicrobial Awareness Week

**DOI:** 10.1017/ash.2022.320

**Published:** 2022-11-18

**Authors:** Bradley J. Langford, Kelly L. Matson, Khalid Eljaaly, Anucha Apisarnthanarak, Pamela L. Bailey, Lindsay MacMurray, Alexandre R. Marra, Kari A. Simonsen, Pranavi Sreeramoju, Priya Nori, Gonzalo M. Bearman

**Affiliations:** 1 Health Protection, Public Health Ontario, Toronto, Ontario, Canada; 2 College of Pharmacy, University of Rhode Island, Providence, Rhode Island, United States; 3 Department of Pharmacy Practice, Faculty of Pharmacy, King Abdulaziz University, Jeddah, Saudi Arabia; 4 Infectious Diseases Division, Thammasat University Hospital, Pathum Thani, Thailand; 5 Infectious Diseases, Prisma Health, Columbia, South Carolina, United States; 6 Society for Healthcare Epidemiology of America, Arlington, Virginia, United States; 7 Department of Internal Medicine, University of Iowa Carver College of Medicine, Iowa City, Iowa, United States; 8 University of Nebraska Medical Center, Omaha, Nebraska, United States; 9 Jefferson Health–Northeast, Philadelphia, Pennsylvania, United States; 10 Department of Medicine, Albert Einstein College of Medicine, Montefiore Medical Center, Bronx, New York, United States; 11 Division of Infectious Diseases, Virginia Commonwealth University Health, Virginia Commonwealth University, Richmond, Virginia, United States

## Abstract

One fundamental strategy to address the public health threat of antimicrobial resistance (AMR) is improved awareness among the public, prescribers, and policy makers with the aim of engaging these groups to act. World Antimicrobial Awareness Week is an opportunity for concerted and consistent communication regarding practical strategies to prevent and mitigate AMR. We highlight 10 ways for antimicrobial stewards to make the most of World Antimicrobial Awareness Week.

Antimicrobial resistance (AMR) endangers health by its effect on morbidity, mortality, hospital length of stay, and healthcare costs.^1^ Currently, global statistics estimate that 1.27 million deaths are directly related to AMR annually.^2^ Moreover, AMR extends beyond increased health risks to agriculture, food security, and socioeconomic development, making it one of the world’s most urgent public health issues.^3^ To bring about needed change, the World Health Organization (WHO) has developed a Global Action Plan on Antimicrobial Resistance. The first of 5 objectives of this plan is “to improve awareness and understanding of antimicrobial resistance through effective communication, education and training.” Awareness is the initial step toward ensuring the continued ability to treat and prevent infectious diseases worldwide.^4^ In this article, we convey key considerations when developing antimicrobial awareness campaigns, with a particular focus on World Antimicrobial Awareness Week, while highlighting relevant work published in *Antimicrobial Stewardship & Healthcare Epidemiology* (ASHE).

## 1. Think globally

### Antimicrobial resistance (AMR) is a serious global health concern

World Antimicrobial Awareness Week (WAAW) is a global event held annually from November 18–24.^5^ WAAW promotes best practices among the public, clinicians, and policy makers to reduce AMR. The WHO encourages the promotion of optimal antimicrobial use and preventive measures targeting AMR, collaborating across sectors and borders, and recognizing the interconnection between humans, animals, plants and the environment, a concept known as One Health.

## 2. Act locally

### Adapt messaging to your audience

Most campaigns have generic messaging targeting the awareness of healthcare workers and the public. But adapting messaging to unique local issues may help close knowledge gaps or address different cultural beliefs related to antibiotics.^6^ Examples may include direct communication of local prescribing rates and antibiograms (if available) to practitioners and public education to reduce antibiotic misuse for viral infections. Countries’ socioeconomics may also influence the messaging, such as minimizing access to antibiotics without a prescription in low- and middle-income countries and reducing sharing of antibiotics or the use of shorter courses in high-income countries.

## 3. Make it personal

While campaigns focusing on the approaching ‘antibiotic apocalypse’ work to capture public attention, qualitative interview data show that these messages are often too sensationalized, which may backfire on their credibility.^7^ Both clinicians and patients view AMR as a geographically and temporally distant phenomenon with a less personal impact on them.^8^ Rather than the more nebulous societal consequences of AMR, antimicrobial awareness campaigns should shift focus to the personal impact of AMR, including the very tangible and current risk of side effects, personal risk of AMR affecting future treatment options, and the perturbations to the microbiome.

## 4. Make the invisible visible

What does AMR look like? The concept of AMR is abstract to many. The exceedingly large projections of the impact of AMR do not tell the personal story of the devastation caused by drug-resistant infections on individual lives. Sharing patient stories can help ‘put a face’ to AMR, making the problem more relatable and inspiring efforts for change.^9,10^ Campaigns like ‘Go Blue for AMR’ include wearing light blue and illuminating local buildings and landmarks in light blue, which can further the visibility of what some have called the ‘silent pandemic.’^11^

## 5. Think beyond the hospital walls and beyond the prescriber

Antimicrobial stewardship was initiated in the hospital setting; however, 80% of antibiotics are used in the community.^12^ Of those antibiotics, 20%–50% are used inappropriately. Primary care providers cite patient pressure for prescribing antibiotics for viral illnesses.^13^ Engaging physicians, veterinarians, and other healthcare professionals and policy makers in AMR awareness is essential to promoting One Health coalitions.^4^ This need is highlighted by the WAAW 2022 theme, ‘Preventing antimicrobial resistance together.’^5^ Professional education and training for students and practitioners in human and animal health should also be established, as should the inclusion of antimicrobial use and resistance in school curricula and media to promote a better understanding of AMR.^4^

Several recent ASHE publications have stressed the importance of broadening the horizon of antimicrobial stewardship awareness beyond prescribers and patients as well as outside the hospital walls. Gullen et al^14^ found that while ambulatory cancer center staff largely knew the term “antimicrobial stewardship,” there were opportunities to improve knowledge about appropriate antibiotic use, particularly among nurses and other clinic staff. Catanzaro et al^15^ highlighted the importance of involving nurses in antimicrobial stewardship using educational modules. Similarly, Manning et al^16^ demonstrated how patient simulations can improve nursing-student awareness of their role in antimicrobial stewardship. Hughes et al^17^ identified several opportunities for improvement in dental antimicrobial stewardship, including improved awareness of and access to evidence-based guidelines, as well as harnessing social comparison to improve prescribing behavior.

## 6. Reframe what the “safe side” is

Antibiotics are often prescribed “just in case” and to “be on the safe side.” In a cross-sectional study using interviews of 90 dentists, 91.2% stated that they prescribed on a ‘just in case’ basis.^17^ In a retrospective cohort study, 205 patients (80.7%) received empiric antibiotic therapy indicated for presumed urinary tract infection despite meeting criteria for asymptomatic bacteriuria.^18^ However, given the known harms of antibiotic therapy, there is an opportunity to reframe messaging regarding “erring on the side of caution” to now mean thoroughly evaluating and monitoring the patient while considering other noninfectious causes before prescribing an antibiotic.

## 7. Meet the public where they are: use creative social media strategies

Social media has facilitated the rapid spread of health-related misinformation,^19^ but at the same time, it provides an instant connection between infectious diseases experts and the public. Social media platforms present not only an opportunity, but a responsibility to share accurate, clear, consistent, and engaging messaging on AMR.^20^ Endless opportunities exist to engage healthcare professionals and the public using creative approaches such as infectious diseases memes, clue-based knowledge assessment quizzes, and personality quizzes.^21^ Antibiotic awareness messaging can be embedded in each engagement strategy, tailored to the captive audience.

## 8. Leverage awareness related to the COVID-19 pandemic

The lessons we learned during the COVID-19 pandemic can also apply to antimicrobial stewardship.^22^

1. *Antibiotics don’t treat viral infections*, whether it be COVID-19 or the common cold. In the United States, ∼44% of outpatient encounters include acute upper-respiratory infections.^23^ Despite the majority of illnesses being caused by viruses, many patients still receive antibiotics, suggesting a need for improved awareness of the risks and benefits of antibiotics in this setting.^24^
2. *Infectious diseases can be devastating*. Unlike COVID-19, AMR is not caused by a single organism and presents a more insidious threat. We need to act quickly to prevent these devastating consequences while we still can. The increased awareness regarding the importance of infection prevention and control during the pandemic should also apply to AMR and may help garner research funding and public-sector support.^25^

## 9. Use evidence-based messaging in awareness campaigns

Antimicrobial awareness messages should be based on scientific evidence. A previously common message to “finish the antibiotic course, even if you are feeling better” was replaced with more nuanced messaging recognizing the growing evidence base for shorter and more tailored courses of therapy.^6^ In addition, behavioral science needs to be incorporated into campaigns and formally evaluate and disseminate findings to help inform future antimicrobial awareness activities. Of 60 campaigns surveyed by the WHO, only 25 were evaluated, and for most of these campaigns, the results were not published.^6^ Wellcome’s Reframing Resistance report provides a list of practical evidence-based suggestions for awareness campaigns, including shifting fear-based messaging to that focusing on personal impact and opportunities for immediate action.^7^ Although there is evidence that fear-based messaging can be effective, it should be combined with empowerment to encourage action rather than apathy.^26^

## 10. Continue antimicrobial awareness activities all year

Antimicrobial awareness should not end after WAAW. Awareness of AMR is necessary but insufficient to change behavior. The impact of awareness campaigns on antibiotic use is mixed, but any impact will not be sustained without ongoing messaging.^27^ Thus, World Antimicrobial Awareness Week should be considered the first step in a long and iterative process, combined with policy, economic, and social strategies to improve antibiotic use.^28^

In conclusion, AMR is a global and universal issue, neither respecting borders nor confined to certain populations. AMR already has a significant impact on health, and if no action is taken, it will become increasingly burdensome. AMR is a solvable issue and awareness of antimicrobial overuse and its consequences is the first step to safeguarding antimicrobials. The 10 concepts discussed in this article can be applied to help maximize antimicrobial awareness efforts as a vital step in ensuring that we can continue to treat and prevent infectious diseases in the years to come.
